# Clonal Expansion of Multidrug-Resistant *Streptococcus dysgalactiae* Subspecies *equisimilis* Causing Bacteremia, Japan, 2005–2021

**DOI:** 10.3201/eid2903.221060

**Published:** 2023-03

**Authors:** Koh Shinohara, Kazunori Murase, Yasuhiro Tsuchido, Taro Noguchi, Satomi Yukawa, Masaki Yamamoto, Yasufumi Matsumura, Ichiro Nakagawa, Miki Nagao

**Affiliations:** Kyoto University Graduate School of Medicine, Kyoto, Japan

**Keywords:** Streptococcus dysgalactiae subspecies equisimilis, bacteria, antimicrobial resistance, streptococci, bacteremia, transposase, Japan, SDSE, streptococci

## Abstract

Incidence of *Streptococcus dysgalactiae* subspecies *equisimilis* (SDSE) bacteremia is increasing in the Kyoto-Shiga region of Japan. We retrospectively analyzed clinical features of SDSE bacteremia and conducted comparative genomic analyses of isolates collected from 146 bacteremia episodes among 133 patients during 2005–2021. Of those patients, 7.7% required vasopressor support, and 7.0% died while in the hospital. The prevalence of isolates resistant to erythromycin, minocycline, and clindamycin increased from 8.6% during 2005–2017 to 21.6% during 2018–2021. Our genomic analysis demonstrated that sequence type 525 and clonal complex 25 were predominant in SDSE isolates collected during 2018–2021. In addition, those isolates had acquired 2 antimicrobial-resistance genes, *erm*B and *tet*M, via Tn*916*-like integrative and conjugative elements (ICEs). Phylogenetic analysis revealed clonal distribution of Tn*916*-like ICEs in SDSE isolates. Our findings suggest that Tn*916*-like ICEs contributed to the emergence and recent increase of multidrug-resistant SDSE bacteremia in this region of Japan.

## Abstract 2

Clones with acquired Tn*916*-like integrative conjugative elements are associated with increased prevalence.

*Streptococcus dysgalactiae* subspecies *equisimilis* (SDSE) is a member of the pyogenic group of streptococci that typically is agglutinated by serum against Lancefield group G or C antigens (rarely A or L antigens) (*1*). Although SDSE has been considered a part of the commensal flora and is much less virulent than *S. pyogenes*, SDSE increasingly has been recognized as a clinically relevant pathogenic bacterium (*2*–*4*). SDSE can cause a broad range of diseases, from milder illnesses such as pharyngitis and skin and soft-tissue infections to severe conditions such as streptococcal toxic shock syndrome (STSS) and necrotizing fasciitis that can resemble infections caused by *S. pyogenes* (*2*,*4*–*6*).

Invasive SDSE infections mainly affect elderly persons with underlying illnesses (*2*,*4*,*6*); fatality rates of 2%–20% have been reported (*4*,*7*). Moreover, multiple countries, including Israel, Denmark, Norway, and Canada, have reported increasing incidence of invasive diseases caused by SDSE or group C or G *Streptococcus* (GCGS) (*8*–*11*). In Japan, a single-center study in Tokyo reported a substantial increase in the age-adjusted incidence of invasive group G *Streptococcus* from 2003–2007 to 2008–2013 (*12*). An aging population with multiple underlying conditions only partially explains those reports (*8*,*12*), and other reasons for the growing prevalence of invasive SDSE infections remain unclear.

SDSE is essentially susceptible to penicillin and other β-lactam antibiotics, but resistance to other antimicrobial agents has emerged. Multiple countries, including the United States, Japan, and Norway, have reported increased prevalence of erythromycin- and clindamycin-resistant isolates (*2*,*5*,*13*). Moreover, recent studies in countries in eastern Asia showed much higher prevalence of resistance to multiple antimicrobial agents, including macrolides, tetracyclines, and lincosamide (*14*,*15*). A multicenter study in China showed resistance rates of 71.4% to erythromycin, 71.4% to clindamycin, and 60.7% to tetracycline (*15*). The prevalent genes responsible for macrolide resistance in those studies were *mef*A/E, *erm*A, and *erm*B (*5*,*13*–*15*); *erm*A and *erm*B are also responsible for clindamycin resistance and typically confer inducible and constitutive resistance.

We conducted a retrospective, multicenter, surveillance study of SDSE bacteremia cases in the Kyoto-Shiga region of Japan. We also performed a comparative genomic analysis of clinical SDSE isolates preserved in 3 hospitals in the region to explore the phylogenetic relationships and emergence of antimicrobial resistance (AMR).

## Materials and Methods

### Surveillance Data Collection

**Figure 1:**
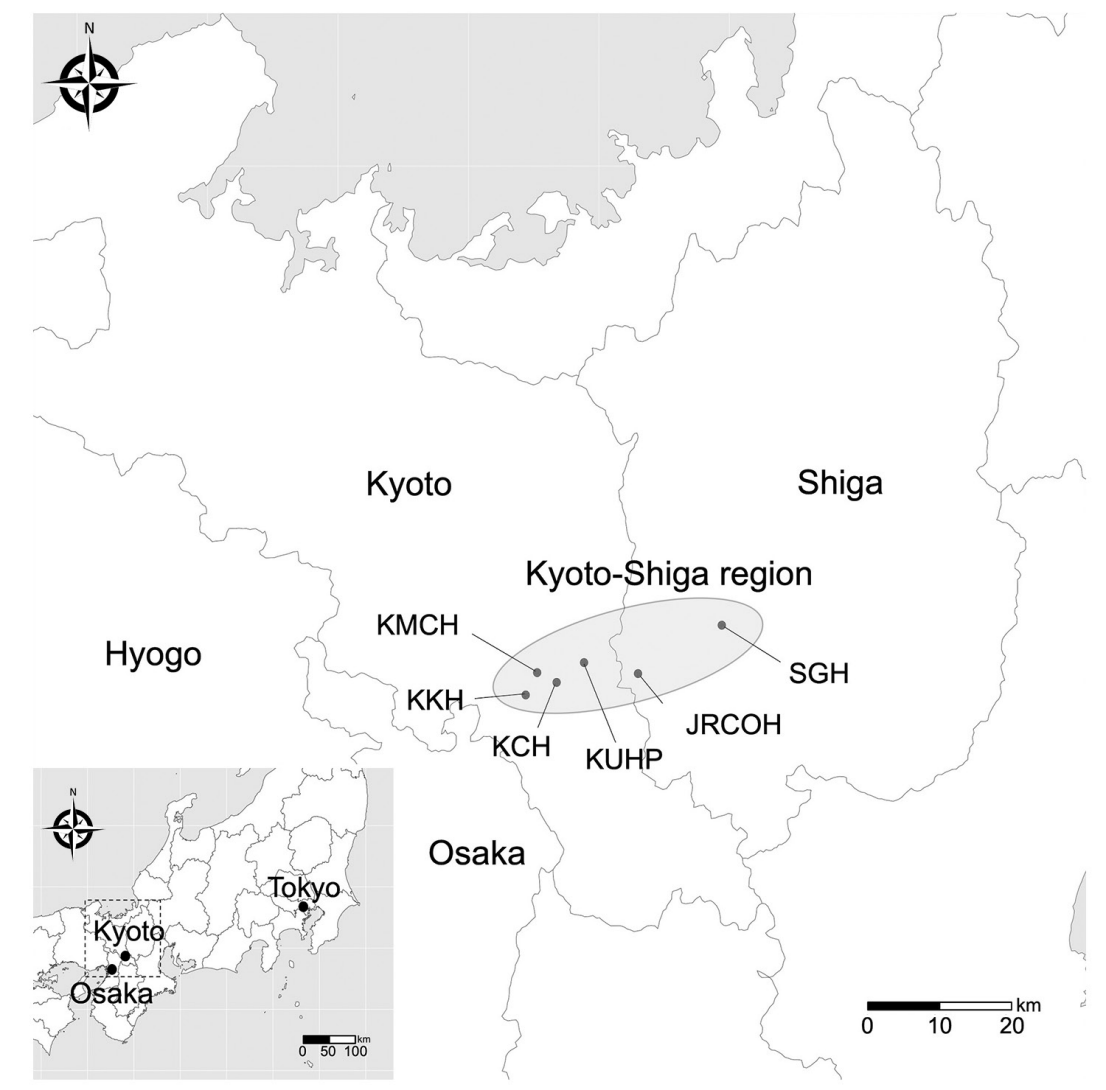
Study area for investigation of clonal expansion of multidrug-resistant *Streptococcus dysgalactiae* subspecies *equisimilis* causing bacteremia, Japan, 2005–2021. Shading indicates the Kyoto-Shiga region and hospitals included in the study. Inset shows study area in Japan. KCH, Kyoto City Hospital; KKH, Kyoto Katsura Hospital; KMCH, Kyoto Min-iren Chuo Hospital; KUHP, Kyoto University Hospital; JRCOH, Japanese Red Cross Otsu Hospital; SGH, Shiga General Hospital.

We collected the annual number of GCGS and SDSE bacteremia cases and hospital admissions during January 2011–December 2020 in 6 hospitals in the Kyoto-Shiga region of Japan. Four hospitals were in Kyoto, Kyoto University Hospital (KUHP), Kyoto City Hospital (KCH), Kyoto Katsura Hospital (KKH), and Kyoto Min-iren Chuo Hospital (KMCH); 2 hospitals were Shiga, Japanese Red Cross Otsu Hospital (JRCOH) and Shiga General Hospital (SGH) (Figure 1). Among the hospitals, 3 changed their methods of identifying β-hemolytic streptococci during the study period, from the biochemical and Lancefield grouping methods to matrix-assisted laser desorption/ionization time-of-flight (MALDI-TOF) mass spectrometry: KUHP in changed in 2016, KCH in 2015, and KMCH in 2019. The other 3 hospitals used the biochemical and Lancefield grouping methods throughout the study period; therefore, we collected the combined incidences of GCGS and SDSE bacteremia.

### Bacterial Isolates

We conducted a microbiological and genomic study using the preserved isolates collected from GCGS and SDSE isolates from the bacteremia cases during 2005–2021 in 3 hospitals: KUHP, KKH, and JRCOH. The bacterial isolates from patients’ blood samples were identified as GCGS when isolates showed large colony size (>0.5 mm), β-hemolysis on 5% sheep blood agar plate after overnight culture, and Lancefield grouping C or G. In some hospitals, GCGS isolates were then identified as SDSE by using phenotypic methods such as API 20 Strep (bioMérieux), BBL Crystal (Becton Dickinson Microbiology Systems), Microscan Walkaway System (Beckman Coulter, Inc.), or MALDI-TOF mass spectrometry. The study comprised 146 SDSE isolates collected during 2005–2021 (Appendix 1). 

Lancefield grouping was performed using the Prolex Streptococcal Grouping Latex Kit (Pro Lab Diagnostics Inc.). Antimicrobial susceptibility testing was conducted by using the MicroScan WalkAway System according to the manufacturers’ instructions or by broth microdilution by using customized dry plates (Eiken Chemical Co., Ltd). Susceptibility results were interpreted following Clinical Laboratory Standards Institute recommendations (*16*). Multidrug-resistant (MDR) was defined as nonsusceptibility to >2 antimicrobial agents; for this study, we refer to MDR as nonsusceptibility to erythromycin, minocycline, and clindamycin.

### Clinical Data Collection

We retrospectively reviewed the charts of patients with preserved isolates. Using clinical records from each episode, we collected the age, sex, dates of illness onset and sample collection, underlying illnesses, receipt of chemotherapy or immunosuppressive agents, signs, symptoms, laboratory findings at the first visit, clinical source of the bacteremia, infection type (community-acquired or nosocomial: nosocomial infections were defined as clinical signs and symptoms occurring >48 hours after admission), infection severity parameters, outcomes, and relapses.

### Whole-Genome Sequencing and Bioinformatic Analysis

We extracted genomic DNA from isolates by using the MagNA Pure 96 DNA and Viral NA Small Volume Kit on a MagNA Pure 96 Instrument (Roche). We prepared a short-read library by using the Illumina DNA Prep Kit (Illumina Inc.) and used NextSeq 1000/2000 P1 Reagent Kit version 3 on the NextSeq platform (Illumina) to generate paired-end reads. We used the Rapid Barcoding Kit (Oxford Nanopore Technologies) to prepare a long-read library from unsheared genomic DNA, which we sequenced by using an R9.4.1 Flow Cell on a MinION device (Oxford Nanopore Technologies). We assembled draft genome sequences from the short-reads and complete genome sequences from the long-reads by using Unicycler pipeline version 0.4.8 with default parameters (*17*). We annotated assembled genomes by using Prokka version 1.14.6 (*17*) with default settings and used annotated genomes for downstream analysis. We confirmed isolates as SDSE by comparing 16S rRNA gene similarity with the subsequent contigs.

We identified multilocus sequence types (MLSTs) from the assembled contigs by using a BLASTn search (https://blast.ncbi.nlm.nih.gov) against the *S. dysgalactiae* MLST database in pubMLST (https://pubmlst.org/organisms/streptococcus-dysgalactiae) and then assigned the MLST to a clonal complex (CC) by using global optimal eBURST (goeBURST). We assigned CCs by a single-locus variation from a founding sequence type (ST). We determined *emm* types via BLAST search against the *emm* sequence database curated by the Centers for Disease Control and Prevention (https://www2.cdc.gov/vaccines/biotech/strepblast.asp). We detected AMR genes by using abricate (https://github.com/tseemann/abricate) and the Comprehensive Antibiotic Resistance Database (https://card.mcmaster.ca) (*18*). We predicted virulence factors of SDSE genomes by using BLASTP against the Virulence Factor Database (http://www.mgc.ac.cn/VFs) (*19*) set A, a core dataset that covers genes associated with experimentally verified virulence factors, and a cutoff E-value of 1E−5.

We submitted the raw Illumina and MinION read data to the International Nucleotide Sequence Database Collaboration (https://www.insdc.org) under BioProject accession no. PRJDB12179. We summarized the assembly statistics and general genomic information (Appendix 1).

### Phylogenetic Analyses

We used Roary version 3.13.0 (*20*) and default parameters to identify the core genomes of the 146 SDSE isolates. We generated a maximum-likelihood phylogenetic tree from the core genome alignment by using RAxML-NG version 1.0.3 and a general time-reversible plus gamma distribution DNA substitution model with 100 bootstrap replicates (*21*). We used FigTree version 1.4.4 (https://github.com/rambaut/figtree) and iTOL version 6 (*22*) to visualize phylogenetic trees.

### Tn*916*-Like Integrative and Conjugative Element Analysis

We extracted sequences of the Tn*916*-like integrative and conjugative elements (ICEs) from the assembled contigs by using a BLASTn search (*23*) against the *Enterococcus faecalis* Tn*916* reference sequence (GenBank accession no. U09422.1). We manually analyzed the region structures by using the Artemis Comparison Tool (*24*).

### Statistical Analyses

We used χ^2^ and Fisher exact tests to compare categorical variables, as appropriate, and used Mann-Whitney U tests to analyze continuous, nonparametric data. To assess the time trends in GCGS/SDSE bacteremia incidence during 2011–2020, we calculated the incidence rate ratio and significance of the data by comparing the later cohort (2016–2020) with the early cohort (2011–2015) and considered a 2-sided p<0.05 statistically significant. We performed all statistical analyses using R version 3.6.0 (The R Foundation for Statistical Computing, https://www.r-project.org).

The Ethics Committee of Kyoto University Graduate School and Faculty of Medicine approved the study (approval no. R3240). The study was conducted in accordance with the principles expressed in the Declaration of Helsinki.

## Results

### Temporal Trends in SDSE Bacteremia

**Figure 2:**
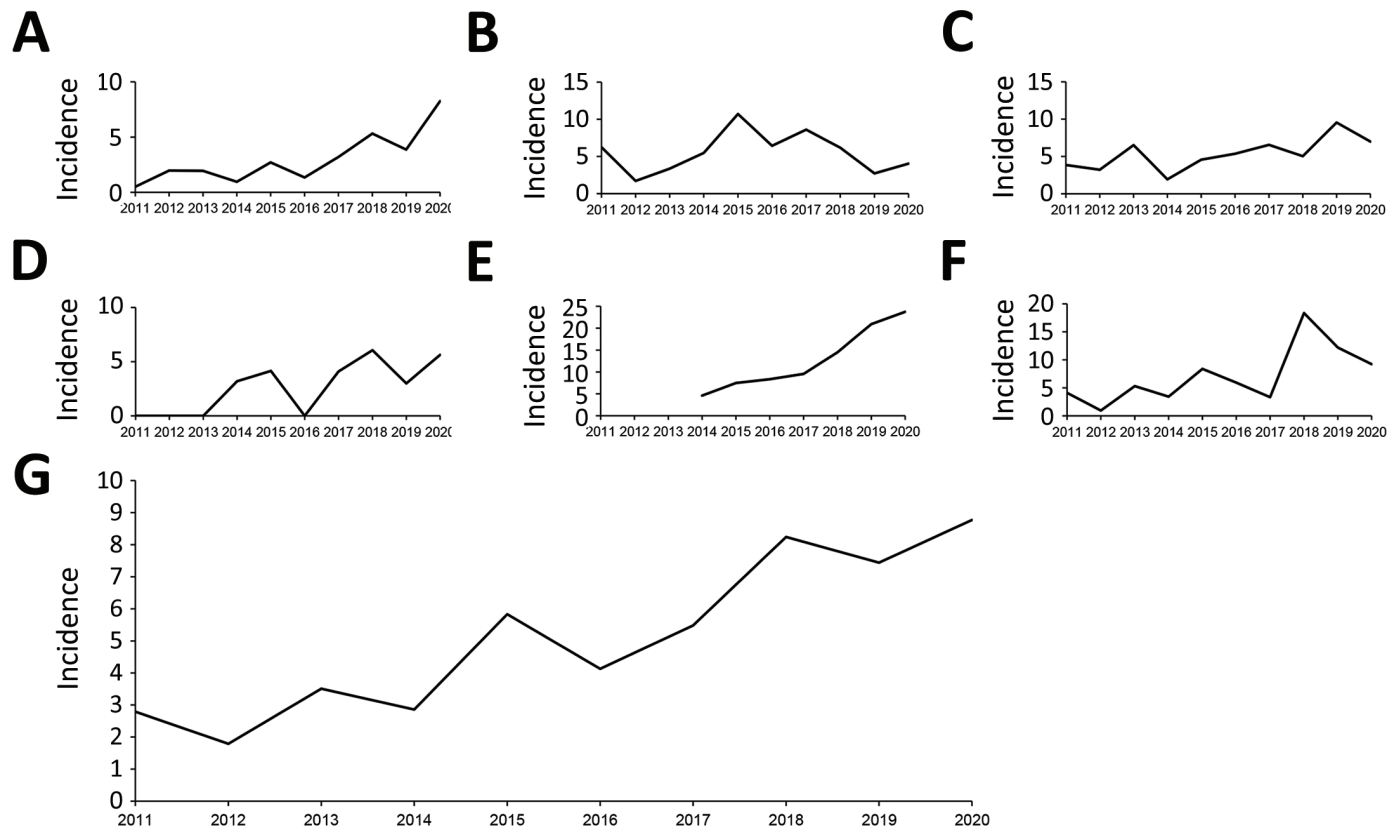
Incidence (cases/10,000 admissions) of multidrug-resistant *Streptococcus dysgalactiae* subspecies *equisimilis* and group C or G *Streptococcus* bacteremia, Kyoto-Shiga region, Japan, 2011–2020. A) Kyoto University Hospital; B) Kyoto City Hospital; C) Japanese Red Cross Otsu Hospital; D) Shiga General Hospital; E) Kyoto Min-iren Chuo Hospital; F) Kyoto Katsura Hospital; G) total for all 6 hospitals included in the study.

The Kyoto-Shiga Region is a metropolitan area in Japan with a population of ≈2.5 million within a range of 70 km. We collected data on 398 episodes of bacteremia caused by GCGS and SDSE in 6 hospitals in the Kyoto-Shiga region during 2011–2020 (Figure 1). We investigated the temporal trend in combined incidence of GCGS and SDSE bacteremia per 10,000 admissions (Figure 2). The incidence of GCGS and SDSE bacteremia increased from 2.77/10,000 admissions in 2011 to 8.77/10,000 admissions in 2020. We noted a statistically significant increase in GCGS and SDSE bacteremia incidence between the first and last 5-year periods (incidence rate ratio = 2.03, p = 0.03). During 2018–2020, the average annual incidence of bacteremia reached 13.3 cases/10,000 admissions in KKH and 19.7 cases/10,000 admissions in KMCH.

### Clinical Characteristics of Patients with SDSE Bacteremia

We investigated 146 SDSE bacteremia episodes by using preserved isolates from 133 patients at KUHP, KKH, and JRCOH. The study included 118 isolates collected from KUHP, KKH, and JRCOH, representing 50.6% (118/233) of all cases from the 3 hospitals during 2011–2020. KUHP preserved consecutive isolates throughout the study period (2005–2021), but JRCOH and KKH did not. In detail, JRCOH preserved consecutive isolates during 2015–2021 but only preserved 1 isolate during 2013–2014. Similarly, KKH preserved 8 of 20 isolates collected in 2021.

Among 133 patients with SDSE bacteremia, most were 80–89 years of age (Table 1). Many patients had underlying conditions, predominantly cerebrovascular disease (28.6%), solid organ cancer (27.1%), and dementia (21.3%). We noted recurrent SDSE bacteremia in 9 patients who had a total of 13 episodes (Appendix 2 Table 1).

**Table 1:** Characteristics of 133 patients with *Streptococcus dysgalactiae* subspecies *equisimilis* bacteremia, Japan, 2005–2021*

**Characteristic**	**Value**
Median age, y (IQR)	81.0 (72.0–88.0)
Age group, y	
<30	1 (0.8)
30–39	4 (3.0)
40–49	5 (3.6)
50–59	6 (4.5)
60–69	12 (9.0)
70–79	29 (21.8)
80–89	50 (37.6)
>90	26 (19.5)
Sex	
M	72 (54.1)
F	61 (45.9)
Medical history	
No underlying conditions	15 (11.3)
Solid organ tumor†	36 (27.1)
History of surgery, n = 36	15 (41.7)
Active malignancy, n = 36‡	22 (61.1)
Metastasis, n = 36	10 (27.8)
Hematologic malignancy	6 (4.5)
Hematopoietic stem cell transplant, n = 6	
Cardiovascular disease	21 (15.8)
Cerebral artery diseases	38 (28.6)
Chronic kidney diseases	27 (20.3)
Diabetes mellitus	22 (16.5)
Dementia	31 (23.3)
Collagen diseases	8 (6.0)
Chronic lung diseases	7 (5.3)
HIV	0
Chemotherapy	12 (9.0)
Immune suppressive therapy	6 (4.5)
Neutropenia, neutrophil count <500 cells/µL	5 (3.8)

*Values are no. (%) except as indicated. For patients with multiple episodes, variables for the first episode are described. †Ten patients with prostate cancer; 8 with cervical cancer; 7 with colon, rectal, or anal cancer; 3 with lung cancer; 2 with stomach cancer; 2 with endometrial cancer; 2 with bladder cancer; 2 with breast cancer; and 1 with cancer categorized as other (esophageal cancer, ovarian cancer, pharyngeal cancer, salivary gland cancer, vaginal cancer, and extramammary Paget’s disease), including duplicates. ‡Treated within 5 years.

Of the 146 episodes in 133 patients, the most common sources of bacteremia were cellulitis (50.7%) or primary bacteremia (18.5%) (Table 2). Of 142 episodes for which detailed data were available, 7 (4.9%) involved STSS and 11 (7.7%) required vasopressor use; of 143 patients, 10 (7.0%) died during hospitalization.

**Table 2:** Clinical manifestations and severity markers for 146 episodes of multidrug-resistant *Streptococcus dysgalactiae* subspecies *equisimilis* bacteremia, Japan, 2005–2021*

**Characteristics**	**No. (%)**
Type of infection	
Community-acquired	136 (93.2)
Nosocomial	10 (6.8)
Clinical source of bacteremia†	
Cellulitis	74 (50.7)
Primary bacteremia without focus	27 (18.5)
Necrotizing fasciitis	10 (6.8)
Vertebral osteomyelitis and discitis	10 (6.8)
Psoas abscess	6 (4.1)
Septic arthritis	11 (7.5)
Infectious endocarditis	4 (2.7)
Urinary tract infection	7 (4.8)
Pneumonia	1 (0.7)
Others‡	14 (9.6)
Clinical characteristics	
Body temperature >38°C, n = 143	100 (69.9)
Mean arterial pressure <80 mm Hg, n = 140	42 (30.0)
Heart rate >90 beats/min, n = 138	83 (60.1)
Disturbance of consciousness, n = 141	54 (38.3)
Severe disease, n = 142	
Streptococcus toxic shock syndrome	7 (4.9)
Vasopressor support required	11 (7.7)
Ventilator support required	6 (4.2)
Admission to intensive care unit required	9 (6.3)
Death	
In-hospital death, n = 143	10 (7.0)
30-d mortality, n = 138	5 (3.6)

*Data include 13 relapse or reinfection episodes among 9 patients (details are available in Appendix 2 Table 1). †Data include >1 instance per patient, including 3 case-patients with cellulitis and septic arthritis; 2 with vertebral osteomyelitis and psoas abscess; and 1 with each of the following co-infections: cellulitis and vertebral osteomyelitis; cellulitis and psoas abscess; cellulitis and urinary tract infection; necrotizing fasciitis and septic arthritis; vertebral osteomyelitis and septic arthritis; psoas abscess and septic arthritis; infective endocarditis and vertebral osteomyelitis; cellulitis and mycotic aneurysm; vertebral osteomyelitis, psoas abscess, and pyogenic lymphadenitis; vertebral osteomyelitis, psoas abscess, and urinary tract infection; vertebral osteomyelitis, septic arthritis, and empyema. ‡Other infections were 3 cases of catheter-related bloodstream infection; 3 cases of decubitus infection; 2 cases of secondary peritonitis; and 1 case each of empyema; surgical site infection; retroperitoneal abscess; pyogenic lymphadenitis; and mycotic aneurysm.

Of 146 SDSE bacteremia episodes, most (136, 93.2%) were community-acquired; 10 (6.8%) were nosocomial (Appendix 2 Table 2). Of the 10 patients with nosocomial episodes, 7 had active malignancy, 5 received chemotherapy, and 3 had febrile neutropenia. Four patients had multiple pathogen infections: 2 episodes were primary bacteremia occurring in patients with neutropenia, the other 2 catheter-related bloodstream infections. In the neutropenic patients with primary bacteremia, *Escherichia coli* or viridans streptococci were isolated from each patient. In the patients with catheter-related bloodstream infections, *Staphylococcus aureus* or *S. epidermidis* was isolated from each patient. Two nosocomial-onset patients died while hospitalized.

### AMR and Virulence Profiles of SDSE Isolates

Of the 146 SDSE isolates identified, 138 were classified as Lancefield group G, 6 as group C, 1 as group A, and 1 as untypeable; 25 STs were represented, and a goeBURST analysis further classified the 25 STs into 7 CCs and 9 singletons. CC17 (34.2%) was the most prevalent, followed by with CC25 (28.1%), ST525 (11.0%), and CC29 (8.9%). Prevalence of CC17, CC25, and CC29 did not change substantially over time, but after ST525 was first isolated in 2016, its prevalence rate increased significantly, from 1.7% (1/58 isolates) during 2005–2017 to 20.0% (15/88 isolates) during 2018–2020 (p = 0.001) (Table 3; Appendix 2 Figure). Conversely, the prevalence of nonmajor CC and ST isolates (other than CC17, CC25, CC29, and ST525) decreased significantly from 27.6% (16/58 isolates) during 2005–2017 to 11.4% (10/88 isolates) during 2018–2020 (p = 0.015) (Appendix 2 Figure). Patients harboring ST525 strains were significantly older than were patients harboring non-ST525 strains (median age 87.0 years vs. 80.5 years; p = 0.007).

**Table 3:** Temporal changes in clonal complexes and sequence types and antimicrobial nonsusceptibility rates of *Streptococcus dysgalactiae* subspecies *equisimilis* bacteremia, Japan, 2005–2021*

Characteristics	No. (%) isolates		p value
	2005–2017, n = 58	2018–2021, n = 88	
CC or ST			
CC17	23 (39.7)	27 (30.7)	0.288
CC25	13 (22.4)	28 (31.8)	0.261
CC29	5 (8.6)	8 (9.1)	1.000
ST525	1 (1.7)	15 (17.1)	0.001
Others	16 (27.6)	10 (11.4)	0.015
Antimicrobial nonsusceptibility			
Erythromycin	16 (27.6)	30 (34.1)	0.469
Minocycline	17 (29.3)	28 (31.8)	0.855
Clindamycin	11 (19.0)	26 (29.5)	0.176
MDR†	5 (8.6)	19 (21.6)	0.042

*CC, clonal complex; MDR, multidrug resistance; ST, sequence type. †Resistant to erythromycin, minocycline, and clindamycin.

We classified *emm*-type isolates into 19 groups (Appendix 2 Table 3). The most prevalent *emm* type was *stG6792* (28.1%), which was predominant in CC17, followed by *stG245* (20.0%), found only in CC25; *stG485* (9.6%), found in CC29 and CC128; and *stG840* (11.0%) found only in ST525.

We assessed AMR rates according to CC and ST (Table 4). No isolates showed nonsusceptibility to penicillin G, ceftriaxone, or meropenem. Nonsusceptibility rates were 31.5% to erythromycin, 30.8% to minocycline, and 25.5% to clindamycin; prevalence of isolates nonsusceptible to those 3 antimicrobial agents increased significantly, from 8.6% (5/58 isolates) during 2005–2017 to 21.6% (19/88 isolates) during 2018–2021 (p = 0.042). Of note, all 16 ST525 isolates were MDR and were resistant to those 3 antimicrobial agents. For CC25, nonsusceptibility rates were 29.3% for erythromycin, 29.3% for clindamycin, and 39.0% for minocycline. Nonsusceptibility rates were much lower for CC17 and CC29: 18.0% for CC17 and 7.7% for CC29 for erythromycin; 4.0% for CC17 and 7.7% for CC29 for minocycline; and 16.0% for CC17 and 7.7% for CC29 for clindamycin. Further AMR gene analysis showed that *erm*B was the most prevalent (20.5% of all isolates) resistance gene for macrolide resistance and was predominantly found in CC25 and ST525 isolates, and *erm*A (8.9%) was mainly found in CC17 isolates (Table 5). In addition, *tet*M was the predominant gene for tetracycline resistance (31.5%), and 23 of 24 MDR isolates possessed both *tet*M and *erm*B.

**Table 4:** Antimicrobial nonsusceptibility rates among *Streptococcus dysgalactiae* subspecies *equisimilis* clonal complexes and sequence types, Japan, 2005–2021*

**CC or ST**	**Penicillin G**	**Cefotaxime**	**Meropenem**	**Erythromycin**	**Minocycline**	**Clindamycin**
CC17, n = 50	0	0	0	9 (18.0)	2 (4.0)	8 (16.0)
CC25, n = 41	0	0	0	12 (29.3)	16 (39.0)	12 (29.3)
ST525, n = 16	0	0	0	16 (100)	16 (100)	16 (100)
CC29, n = 13	0	0	0	1 (7.7)	1 (7.7)	1 (7.7)
Others, n = 26	0	0	0	8 (30.8)	10 (38.5)	0
Total, n = 146	0	0	0	46 (31.5)	45 (30.8)	37 (25.5)

*Values are no. (%) isolates. CC, clonal complex; ST, sequence type.

**Table 5:** Prevalence of antimicrobial resistance determinant genes and Tn*916*-like integrative and conjugative elements among *Streptococcus dysgalactiae* subspecies *equisimilis* clonal complexes and sequence types, Japan, 2005–2021*

CC or ST	*erm*B	*erm*A	*mef*(A/E)	*tet*M	*tet*L	Tn*916*-like ICE
CC17, n = 50	2 (4.0)	7 (14.0)	0	2 (4.0)	0	2 (4.0)
CC25, n = 41	12 (29.3)	0	0	18 (43.9)	0	17 (41.5)
ST525, n = 16	16 (100)	0	0	16 (100)	0	16 (100)
CC29, n = 13	0	1 (7.7)	0	0	0	0
Others, n = 26	0	5 (19.2)	5 (19.2)	10 (38.5)	2 (7.7)	3 (11.5)
Total, n = 146	30 (20.5)	13 (8.9)	5 (3.4)	46 (31.5)	2 (1.4)	38 (26.0)

*Values are no. (%) isolates. CC, clonal complex; ICE, integrative and conjugative element; ST, sequence type.

We assessed prevalence of the virulence factor–associated genes according to CC (Appendix 2 Table 4). All SDSE isolates contained virulence factor–associated genes, including *fbp54*, *lmb*, *scpA/scpB*, *slo*, *ska*, and *hasC*. Prevalence of other virulence-related genes, such as *sda*, *speG*, and pilus island 1–associated genes, differed between CCs. *speG* was significantly associated with STSS (p = 0.026). However, other virulence genes did not have statistically significant correlation with severe disease (defined as STSS, necrotizing fasciitis, or need for vasopressors) or in-hospital death (Appendix 2 Table 4).

### Phylogenic Analysis of SDSE Isolates

**Figure 3:**
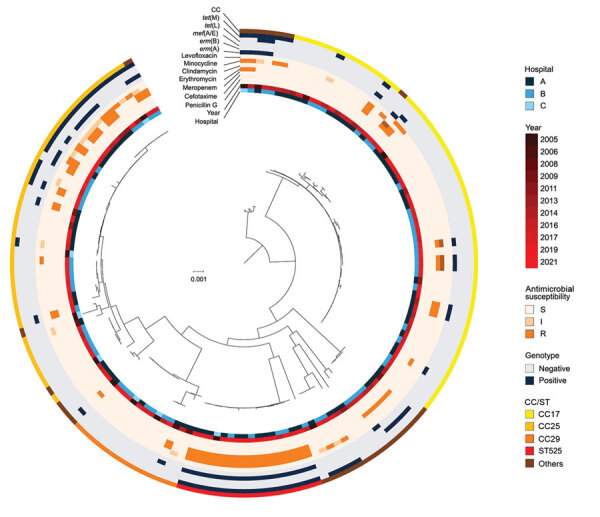
Phylogenetic tree of multidrug-resistant *Streptococcus dysgalactiae* subspecies *equisimilis* causing bacteremia, Japan, 2005–2021. From the center out, rings indicate hospital site, year of isolation, antimicrobial resistance, genotypes, and CC for each isolate. Scale bar indicates nucleotide substitutions per site. CC, clonal complex; I, intermediate; R, resistant; S, susceptible; ST, sequence type.

We visualized a phylogenetic tree of the SDSE isolates based on the 39,258 concatenated single nucleotide polymorphisms of the core genome (Figure 3). Phylogenetic analysis of 146 SDSE strains from Japan revealed no geographic relationships among specific clades or relationships with the year of isolation. However, the CCs reflected phylogenetic relationships, and the phylogenetic tree showed the clonal accumulation of clindamycin, minocycline, and erythromycin resistance in the ST525 clade and a part of the CC25 clade. The isolates possessed both *tet*M and *erm*B AMR genes.

### Structure and Genetic Characterization of Tn*916*-Like ICEs on SDSE Genome

**Figure 4:**
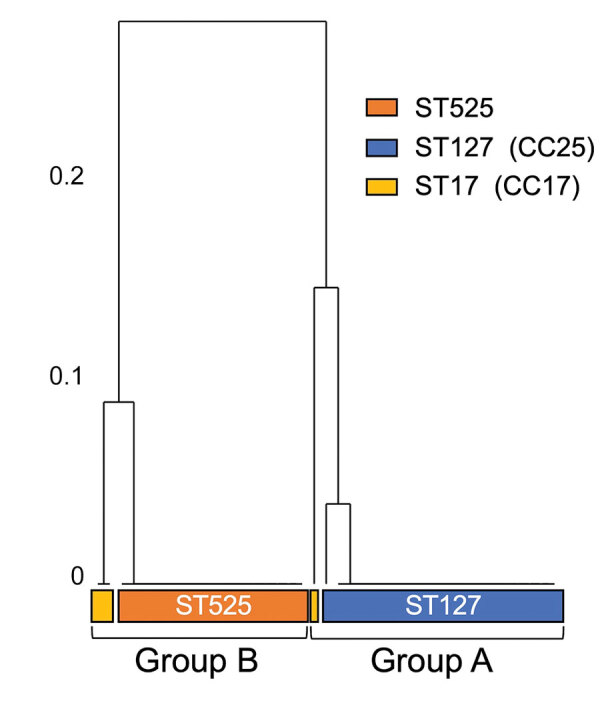
Phylogenetic relationship of group A and group B multidrug-resistant *Streptococcus dysgalactiae* subspecies *equisimilis* causing bacteremia, Japan, 2005–2021. Numbers on left of tree indicate the distance of the clusters. CC, clonal complex; ST, sequence type.

**Figure 5:**
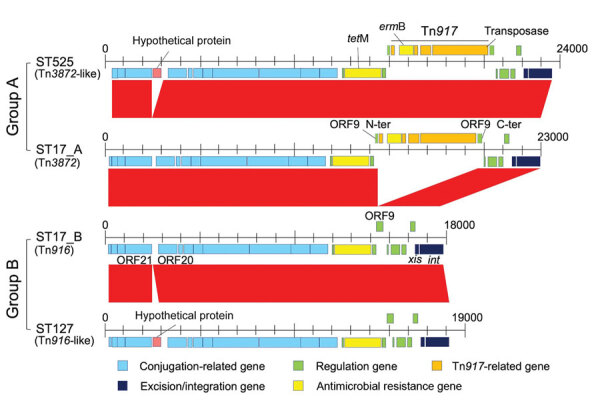
Hypothetical protein substitutions for group A and group B multidrug-resistant *Streptococcus dysgalactiae* subspecies *equisimilis* causing bacteremia, Japan, 2005–2021. CC, clonal complex; ORF, open reading frame; ST, sequence type.

**Figure 6:**
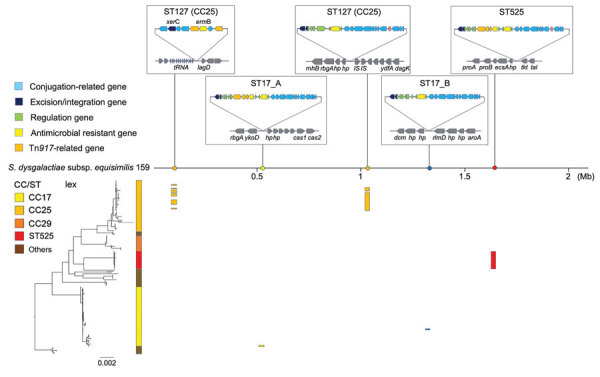
Phylogenetic relationships between clonal complexes of multidrug-resistant *Streptococcus dysgalactiae* subspecies *equisimilis* causing bacteremia, Japan, 2005–2021. Phylogenetic tree is shown in the lower left corner. Scale bar indicates nucleotide substitutions per site. CC, clonal complex; Mb, megabase; ST, sequence type.

To investigate the genetic elements contributing to the clonal accumulation of *tet*M and *erm*B, we performed further sequence analysis by using the complete genome sequences. The *tet*M and *erm*B genes were located on the Tn*916*-like ICEs inserted into the chromosomal backbone. The genomes of 35 (24.0%) of the 146 strains harbored Tn*916*-like ICEs (Table 5). The dendrogram based on prevalence of coding sequences in the elements showed that SDSE-associated Tn*916*-like ICEs were mainly divided into 2 distinct groups (Figure 4). Group A included all 16 ST525 isolates and 1 ST17 (CC17) isolate (Figure 5); group B included all 17 ST127 (CC25) isolates and 1 ST17 isolate (Figures 5, 6). 

We conducted a further detailed structural and sequence comparison of the Tn*916*-like ICEs. In group B, ST17_B had an identical structure to the original Tn*916*, and a hypothetical protein was inserted on the intergenic region between open reading frame (ORF) 20 and ORF21 in all ST127 isolates (Figures 5, 6). In group A, ST17_A had an identical structure to Tn*3872*, which carries Tn*917* with *erm*B inserted in the form of a splitting ORF9 on Tn*916*, and all ST525 isolates had an additional hypothetical protein inserted similar to those in the ST127 isolates (Figures 5, 6). In the ST127 isolates with *erm*B on a sequence region other than Tn*916*-like ICE, *erm*B was sandwiched between 2 IS*1216E* genes and inserted into a specific site (Figure 5). The Tn*916*-like ICEs were clonally distributed in a phylogenic tree; each type of Tn*916*-like ICE showed the distribution pattern along with phylogenetic relationships and specific insertion sites on the genome (Figure 6). To further investigate Tn*3872*-like ICE in ST525, we performed a BLASTn search against the National Center for Biotechnology Information database using parameters of >90% query coverage and >95% identification. We found almost identical genetic structure with highly conserved sequences in other streptococci and nonstreptococci bacteria (Appendix 2 Table 5).

## Discussion

We investigated the genomic characteristics of the SDSE isolates causing increased prevalence of bloodstream infections in the Kyoto-Shiga region of Japan. Our results indicate a recent replacement of the major SDSE CC in this region and increases in isolates that are nonsusceptible to erythromycin, minocycline, and clindamycin. The increased nonsusceptibility rates are mainly because of the emergence of ST525 strains harboring Tn*916*-like ICEs carrying both *tet*M and *erm*B.

The characteristics of SDSE-derived invasive diseases in this study were generally the same as those previously reported (*2*,*4*,*6*,*7*). SDSE predominantly affected elderly patients with underlying diseases, usually skin and soft tissue infections or primary bacteremia, and were sometimes associated with life-threatening STSS or necrotizing fasciitis. Our investigation of clinical information on patients with SDSE bacteremia revealed that 7.7% of patients required vasopressor support and intensive care unit admission, and 7.0% of patients died in the hospital. Those mortality rates indicate the relatively high severity of invasive SDSE disease and are similar to those from previous reports (*4*,*7*), even considering the increasing incidence of SDSE bacteremia. As the global population ages, those findings indicate an increasing burden of invasive SDSE disease in clinical settings. 

Invasive SDSE infections can also contribute to nosocomial onset (*2*,*25*), but detailed clinical features of nosocomial cases are unclear. Our study revealed that nosocomial SDSE infections mainly affected patients with malignant disease who were undergoing chemotherapy, as shown in the clinical data for 3 of 10 patients with nosocomial SDSE who had febrile neutropenia (Appendix 2 Table 1). Compared with *S. pyogenes* and *S. agalactiae*, SDSE can be carried persistently in the throat (*26*). Nosocomial infections, including those in patients with febrile neutropenia, might be associated with a long duration of SDSE carriage on the skin and in oral mucosa, throat, and gastrointestinal tracts.

Multiple countries have reported increased incidences of invasive SDSE disease (*8*,*9*,*11*,*27*,*28*). One reason for these increases might be the increase in aging populations, as previously reported (*2*,*5*,*12*,*14*). During 2011–2020, the aging population of Kyoto City increased; the percentage of persons >65 years of age increased by 21.9% and of persons >80 years of age by 41.9% (*29*). Shiga Prefecture is adjacent to Kyoto Prefecture and constitutes the Kyoto metropolitan area (Figure 1), and active commuting between the prefectures could contribute to further spread of SDSE in the region. In this study, KKH and KMCH showed higher incidences of SDSE bacteremia than the other 4 hospitals. KKH and KMCH had higher proportions of inpatients >70 years of age (66.1% for KKH and KMCH vs. 51.6% for the other 4 hospitals). This rapid increase in the older population might have affected the increasing prevalence of SDSE bacteremia. In addition, the trend in antimicrobial drug prescriptions in Japan is concerning. The proportion of oral macrolide and third-generation cephalosporin consumption to total oral antimicrobial drug consumption is greater in Japan than in Europe or the United States (*30*). Therefore, high selective pressure by macrolides might be contributing to the emergence of macrolide-resistant SDSE, which acquired the resistance gene through mobile genetic elements, such as Tn*916*-like ICEs. The possible long-term persistent carriage of SDSE could increase the antimicrobial selective pressure (*26*), leading to the further selection of resistant strains.

Our genomic analysis indicated that the increased prevalence of MDR SDSE isolates was mainly because of emerging ST525 strains, which uniformly had Tn*3872*-like ICE, a Tn*916*-like element with the insertion of Tn*917* (Figure 6). Increasing evidence suggests that mobile genetic elements, including Tn*916*-like ICEs, are involved in disseminating AMR genes among *S. pneumoniae*, *S. pyogenes*, and *S. agalactiae*, either by clonal expansion or horizontal gene transfer (*31*–*33*). However, the genetic background of macrolide and lincosamide resistance dissemination in SDSE is unclear. A recent whole-genome sequencing study revealed that ICEs carried resistance genes such as *erm*A and *erm*B (*13*). The authors reported that those ICEs exhibited remarkable intraspecies and interspecies similarities, suggesting possible dissemination of resistance genes via conjugative transfer of the ICEs. Phylogenetic analysis showed shorter genetic distances of isolates with Tn*916*-like ICEs in the ST525 and CC25 clades, implying clonal expansion of those isolates. Although we found no evidence of intraspecies or interspecies transfer of Tn*916*-like ICEs among SDSE, macrolide-resistant SDSE could become widespread, as seen with other streptococci (*31*–*33*). Also, the Tn*3872*-like ICEs found in the ST525 were widely distributed in other bacteria, including other streptococci and enterococci (Appendix 2 Table 5), but not in any *S. dysgalactiae* genomes available in public databases. Although another study reported 1 SDSE isolate with Tn*3872*-like ICE, those elements lacked a transposase Tn*4430* in Tn*917* (*13*).

The first limitation of our study is the retrospective design, which only enabled us to use preserved isolates. Although selection bias among the preserved isolates might have affected the incidence of AMR isolates and the epidemiology of AMR genes, the multicenter study and detailed genomic investigation enabled us to assess regional clonal dynamics, especially the dynamics of ICEs carrying AMR genes. The second limitation was that the strain identification methods, such as MALDI-TOF mass spectrometry and Lancefield group typing, varied depending on the facility or year in which the strain was isolated. The strain identification methods might have influenced estimation of SDSE bacteremia incidence because other *Streptococcus* species belonging to Lancefield group C or G and non-GCGS SDSE have been reported (*1*,*34*,*35*). However, previous studies revealed that SDSE causes most GCGS infections in humans (*2*,*4*), and few SDSE isolates represented groups other than C or G in our study. Considering those findings, we believe that estimation of the temporal trend in our study is generally reliable.

In conclusion, our study showed an increasing incidence of SDSE bacteremia in the Kyoto-Shiga region of Japan over the past decade. In addition, clonal expansion of ST525 strains carrying *erm*B and *tet*M on Tn*916*-like ICEs contributed to the emergence of MDR SDSE strains. Continuous surveillance, including whole-genome sequencing, is needed to clarify and predict trends in MDR SDSE strains associated with Tn*916*-like ICEs.

Appendix 1 Strains used for molecular study of clonal expansion of multidrug-resistant *Streptococcus dysgalactiae* subsp. *equisimilis* causing bacteremia, Japan, 2005–2021.

Appendix 2 Additional information on clonal expansion of multidrug-resistant *Streptococcus dysgalactiae* subsp. *equisimilis* causing bacteremia, Japan, 2005–2021.
