# Supplementary material for: Clonal Expansion of Multidrug-Resistant Streptococcus dysgalactiae Subspecies equisimilis Causing Bacteremia, Japan, 2005–2021
Source: Emerg Infect Dis. 2023 Mar;29(3):528–39. doi: 10.3201/eid2903.221060 (PMC9973691; doi:10.3201/eid2903.221060)
Supplement: Appendix 2 — Additional information on clonal expansion of multidrug-resistant Streptococcus dysgalactiae subsp. equisimilis causing bacteremia, Japan, 2005–2021. [file 22-1060-Techapp-s2.pdf]

Article DOI: https://doi.org/10.3201/eid2903.221060

# Clonal Expansion of Multidrug-Resistant *Streptococcus dysgalactiae* Subspecies *equisimilis* Causing Bacteremia, Japan, 2005–2021

## Appendix 2

**Appendix 2 Table 1**. Clinical characteristics of the patients with multiple episodes of *Streptococcus dysgalactiae* subsp. *equisimilis* bacteremia, Japan, 2005–2021*

| Case no. | Age, y/sex | Underlying disease | Indwelling devices | Episode no. | Date | Focus | ST | Antimicrobial drug duration, d |
|---|---|---|---|---|---|---|---|---|
| 1 | 51/F | Active cervical cancer, post surgery, without metastasis | N | 1 | Mar 2012 | Cellulitis, buttocks | ST671 | 14 |
| | | | | 2 | Jan 2013 | Cellulitis, leg and foot | ST127 | 12 |
| 2 | 91/M | Diabetes mellitus with mild diabetic nephropathy, hypertension | Urethral catheter | 1 | May 2018 | CAUTI | ST17 | 15 |
| | | | | 2 | Feb 2020 | CAUTI | ST17 | 17 |
| | | | | 3 | Mar 2021 | CAUTI | ST525 | 14 |
| 3 | 85/M | Congestive heart failure, hypertension | Cardiac pacemaker | 1 | Jul 2019 | Vertebral osteomyelitis, psoas abscess, cervical lymph node adenopathy | ST525 | 58 |

| Case no. | Age, y/sex | Underlying disease | Indwelling devices | Episode no. | Date | Focus | ST | Antimicrobial drug duration, d |
|---|---|---|---|---|---|---|---|---|
| | | | | 2 | Apr 2020 | Primary bacteremia without focus | ST525 | 26 |
| 4 | 74/M | Hemodialysis-dependent end-stage renal disease, Prostate cancer (post surgery) without metastasis, hypertension | N | 1 | Jun 2020 | Primary bacteremia without focus | ST127 | 118 |
| | | | | 2 | Dec 2020 | Vertebral osteomyelitis | ST17 | not finished due to chronic suppression |
| 5 | 83/F | Cervical cancer (post radiation therapy) without metastasis | Total hip replacement | 1 | Oct 2016 | Cellulitis, left extremity | ST34 | 57 |
| | | | | 2 | Jan 2018 | Cellulitis, left extremity | ST34 | 18 |
| 6 | 44/M | Diabetes mellitus with diabetic nephropathy | N | 1 | Apr 2017 | Cellulitis, left extremity | ST17 | 16 |
| | | | | 2 | Dec 2017 | Cellulitis, right extremity | ST628 | 16 |
| 7 | 81/M | Chronic kidney disease, aplastic anemia; immunosuppression via cyclophosphamide | N | 1 | Sep 2018 | Cellulitis, right extremity | ST17 | 17 |
| | | | | 2 | Dec 2018 | Cellulitis, right extremity | ST17 | 14 |
| 8 | 78/M | Cerebral infarction | N | 1 | Feb 2019 | Cellulitis, left extremity | ST25 | 15 |
| | | | | 2 | Jun 2019 | Cellulitis, left extremity | ST25 | 15 |

| Case no. | Age, y/sex | Underlying disease | Indwelling devices | Episode no. | Date | Focus | ST | Antimicrobial drug duration, d |
|---|---|---|---|---|---|---|---|---|
| | | | | 3 | Aug 2019 | Cellulitis, left extremity | ST25 | 14 |
| | | | | 4 | Dec 2020 | Cellulitis, left extremity | ST25 | 15 |
| | | | | 5 | Jun 2021 | Cellulitis, left extremity | ST25 | 8 |
| 9 | 79/F | Anal canal cancer without metastasis, not treated within 5 years | N | 1 | Jan 2020 | Cellulitis, right extremity | ST17 | 17 |
| | | | | 2 | Mar 2020 | Cellulitis, right extremity | ST17 | 43 |

*CAUTI, catheter-associated urinary tract infection; ST, sequence type.

**Appendix 2 Table 2.** Patient demographics and clinical manifestations of 10 patients with nosocomial *Streptococcus dysgalactiae* subsp. *equisimilis* bacteremia, Japan, 2005–2021

| Demographic and clinical data | No. (%) |
|---|---|
| Median age, y (IQR) | 70.5 (67.5– 78.8) |
| Age group, y | |
| <50 | 0 |
| 50–59 | 1 (10.0) |
| 60–69 | 3 (30.0) |
| 70–79 | 4 (40.0) |
| 80–89 | 1 (10.0) |
| ≥90 | 1 (10.0) |
| Sex | |
| M | 8 (80.0) |
| F | 2 (20.0) |
| Medical history | |
| Malignant diseases | 7 (70.0) |
| Hematological malignancy | 1 (10.0) |
| Solid organ tumor | 6 (60.0) |
| Cardiovascular diseases | 2 (20.0) |
| Cerebral artery diseases | 3 (30.0) |
| Chronic kidney diseases | 1 (10.0) |
| Diabetes mellitus | 3 (30.0) |
| Dementia | 1 (10.0) |
| Chronic lung diseases | 0 |
| Collagen diseases | 0 |
| HIV | 0 |
| Chemotherapy | 5 (50.0) |
| Immune suppressive therapy | 1 (10.0) |
| Neutropenia | 3 (30.0) |
| Clinical source of bacteremia, including duplicates* | |
| Cellulitis | 2 (20.0) |
| Primary bacteremia without focus | 4 (40.0) |
| Necrotizing fasciitis | 0 |
| Osteomyelitis and discitis | 1 (10.0) |
| Psoas abscess | 1 (10.0) |

| Demographic and clinical data | No. (%) |
|---|---|
| Septic arthritis | 0 |
| Infectious endocarditis | 0 |
| Urinary tract infection | 0 |
| Pneumonia | 0 |
| Catheter related blood stream infection | 3 (30.0) |
| Multiple pathogen infection | 4 (40.0) |
| *Escherichia coli* | 1 (10.0) |
| *Staphylococcus aureus* | 1 (10.0) |
| Viridans streptococci | 1 (10.0) |
| *Staphylococcus epidermidis* | 1 (10.0) |
| Clinical characteristics† | |
| Temperature $\geq$38°C | 8 (80.0) |
| Mean arterial pressure <80 mm Hg, n = 9 | 5 (55.6) |
| Pulse rate >90 beats/min, n = 9 | 5 (62.5) |
| Disturbance of consciousness | 6 (60.0) |
| Severe disease | |
| Streptococcus toxic shock syndrome | 0 |
| Vasopressor support required | 0 |
| Ventilator support required | 0 |
| Admission to intensive care unit required | 0 |
| Death | |
| In-hospital death | 2 (20.0) |
| 30-d mortality | 1 (10.0) |

*Vertebral osteomyelitis and psoas abscess, 1 case.

†Data on blood pressure was missed in 1 case-patient, and data on pulse rate was missed in 2 case-patients.

**Appendix 2 Table 3.** Correlation with *emm* type and clonal complex among *Streptococcus dysgalactiae* subsp. *equisimilis* isolates causing bacteremia, Japan, 2005–2021*

| *emm* | Clonal complex | | | | | | | Sequence type | | | | | | | | | Total no. |
|---|---|---|---|---|---|---|---|---|---|---|---|---|---|---|---|---|---|
| type | 17 | 25 | 29 | 34 | 146 | 15 | 128 | 129 | 471 | 70 | 8 | 285 | 674 | 675 | 677 | 525 | (%) |
| *stG6792* | 40 | 1 | 0 | 0 | 0 | 0 | 0 | 0 | 0 | 0 | 0 | 0 | 0 | 0 | 0 | 0 | 41 (28.1) |
| *stG245* | 0 | 29 | 0 | 0 | 0 | 0 | 0 | 0 | 0 | 0 | 0 | 0 | 0 | 0 | 0 | 0 | 29 (19.9) |
| *stG840* | 0 | 0 | 0 | 0 | 0 | 0 | 0 | 0 | 0 | 0 | 0 | 0 | 0 | 0 | 0 | 16 | 16 (11.0) |
| *stG485* | 0 | 0 | 10 | 0 | 0 | 0 | 4 | 0 | 0 | 0 | 0 | 0 | 0 | 0 | 0 | 0 | 14 (9.6) |
| *stG6* | 0 | 2 | 0 | 0 | 3 | 0 | 0 | 0 | 0 | 0 | 0 | 0 | 0 | 0 | 0 | 0 | 5 (3.4) |
| *stG166B* | 0 | 5 | 0 | 0 | 0 | 0 | 0 | 0 | 0 | 0 | 0 | 1 | 0 | 0 | 0 | 0 | 6 (4.1) |
| *stG653* | 3 | 0 | 0 | 0 | 0 | 0 | 0 | 0 | 0 | 0 | 0 | 0 | 0 | 0 | 0 | 0 | 3 (2.1) |
| *stG2078* | 3 | 0 | 0 | 0 | 0 | 0 | 0 | 0 | 0 | 0 | 0 | 0 | 0 | 0 | 0 | 0 | 3 (2.1) |
| *stG10* | 0 | 0 | 0 | 0 | 0 | 4 | 0 | 0 | 1 | 0 | 0 | 0 | 0 | 0 | 0 | 0 | 5 (3.4) |
| *stG480* | 0 | 1 | 0 | 0 | 0 | 2 | 0 | 0 | 0 | 0 | 0 | 0 | 0 | 0 | 0 | 0 | 3 (2.1) |
| *stG4974* | 2 | 0 | 0 | 0 | 0 | 0 | 0 | 0 | 0 | 0 | 0 | 0 | 0 | 0 | 0 | 0 | 2 (1.4) |
| *stC1400* | 0 | 0 | 0 | 4 | 0 | 0 | 0 | 0 | 0 | 0 | 0 | 0 | 0 | 0 | 0 | 0 | 4 (2.7) |
| *stC36* | 1 | 0 | 0 | 0 | 0 | 0 | 0 | 0 | 0 | 0 | 1 | 0 | 0 | 0 | 0 | 0 | 2 (1.4) |
| *stG652* | 1 | 1 | 0 | 0 | 0 | 0 | 0 | 0 | 0 | 1 | 0 | 0 | 1 | 0 | 1 | 0 | 5 (3.4) |
| *stG5420* | 0 | 1 | 0 | 0 | 0 | 0 | 0 | 0 | 0 | 0 | 0 | 0 | 0 | 0 | 0 | 0 | 1 (0.7) |
| *stC74a* | 0 | 0 | 3 | 0 | 0 | 0 | 0 | 0 | 0 | 0 | 0 | 0 | 0 | 0 | 0 | 0 | 3 (2.1) |
| *stC6979* | 0 | 0 | 0 | 0 | 0 | 0 | 0 | 1 | 0 | 0 | 0 | 0 | 0 | 0 | 0 | 0 | 1 (0.7) |
| *stG2574* | 0 | 1 | 0 | 0 | 0 | 0 | 0 | 0 | 0 | 0 | 0 | 0 | 0 | 1 | 0 | 0 | 2 (1.4) |
| *st*C15 | 0 | 0 | 0 | 1 | 0 | 0 | 0 | 0 | 0 | 0 | 0 | 0 | 0 | 0 | 0 | 0 | 1 (0.7) |
| Total no. | 50 | 41 | 13 | 5 | 3 | 6 | 4 | 1 | 1 | 1 | 1 | 1 | 1 | 1 | 1 | 16 | 146 |
| (%) | (34.2) | (28.1) | (8.9) | (3.4) | (2.1) | (4.1) | (2.7) | (0.7) | (0.7) | (0.7) | (0.7) | (0.7) | (0.7) | (0.7) | (0.7) | (11.0) | |

**Appendix 2 Table 4.** Prevalence of virulence factors according to the clonal complex or sequence type among *Streptococcus dysgalactiae* subsp. *equisimilis* isolates causing bacteremia, Japan, 2005–2021

| | | Clonal complex or sequence type, no. (%) | | | | | | Severe disease | | | |
|---|---|---|---|---|---|---|---|---|---|---|---|
| Category, virulence factor | Assoc. gene | CC17 | CC25 | CC29 | ST525 | Others | Total | STSS | NF | Vasopressor need | In-hospital mortality |
| Adherence | | N = 50 | N = 41 | N = 13 | N = 16 | N = 24 | N = 146 | | | N = 142 | N = 143 |
| Fibronectin binding protein | *fbp54* | 50 (100) | 41 (100) | 13 (100) | 16 (100) | 26 (100) | 146 (100) | NA | NA | NA | NA |
| | *GBS_RS03565* | 50 (100) | 24 (58.5) | 13 (100) | 16 (100) | 11 (42.3) | 114 (78.1) | 5/114 (p = 0.648) | 10/114 (p = 0.118) | 8/112 (p = 0.452) | 8/112 (p = 1.000) |
| Pilus island 1 | *GBS_RS03570* | 50 (100) | 24 (58.5) | 13 (100) | 16 (100) | 11 (42.3) | 114 (78.1) | 5/114 (p = 0.648) | 10/114 (p = 0.118) | 8/112 (p = 0.452) | 8/112 (p = 1.000) |
| | *GBS_RS03585* | 48 (96.0) | 24 (58.5) | 13 (100) | 16 (100) | 11 (42.3) | 112 (76.7) | 5/112 (p = 0.665) | 10/112 (p = 0.117) | 8/110 (p = 0.483) | 8/110 (p = 1.000) |
| Laminin binding protein | *lmb* | 50 (100) | 41 (100) | 13 (100) | 16 (100) | 26 (100) | 146 (100) | NA | NA | NA | NA |
| C5a peptidase | *scpA/scpB* | 50 (100) | 41 (100) | 13 (100) | 16 (100) | 26 (100) | 146 (100) | NA | NA | NA | NA |
| Exotoxin | | | | | | | | | | | |
| Streptococcal pyrogenic exotoxin | *speG* | 0 (0) | 0 (0) | 13 (100) | 16 (100) | 15 (57.7) | 44 (30.1) | 5/44 (p = 0.026) | 5/44 (p = 0.169) | 4/43 (p = 869) | 5/42 (p = 0.158) |
| Streptolysin O | *slo* | 50 (100) | 41 (100) | 13 (100) | 16 (100) | 26 (100) | 146 (100) | NA | NA | NA | NA |
| Exoenzyme | | | | | | | | | | | |
| Streptokinase | *ska* | 50 (100) | 41 (100) | 13 (100) | 16 (100) | 26 (100) | 146 (100) | NA | NA | NA | NA |
| | *sda* | 1 (2.0) | 5 (12.2) | 0 (0) | 0 (0) | 1 (3.1) | 7 (4.8) | 0/7 (p = 1.000) | 0/7 (p = 1.000) | 0/7 (p = 1.000) | 0/7 (p = 1.000) |
| Immune modulation | | | | | | | | | | | |

| Category, virulence factor | Assoc. gene | Clonal complex or sequence type, no. (%) CC17 | CC25 | CC29 | ST525 | Others | Total | Severe disease STSS | NF | Vasopressor need | In-hospital mortality |
|---|---|---|---|---|---|---|---|---|---|---|---|
| Hyaluronic acid capsule | *hasC* | 50 (100) | 41 (100) | 13 (100) | 16 (100) | 32 (100) | 146 (100) | NA | NA | NA | NA |

*CC, clonal complex; NF, necrotizing fasciitis; SSTS, streptococcal toxic shock syndrome; ST, sequence type.

**Appendix 2 Table 5.** Comparison of Tn*3872*-like integrative conjugative elements among other bacterial species and *Streptococcus dysgalactiae* subsp. *equisimilis* isolates causing bacteremia, Japan, 2005–2021*

| Species | No. of alignments, total = 33 | % Query cover |
|---|---|---|
| *Streptococcus agalactiae* | 6 | 97 |
| *Streptococcus pneumoniae* | 13 | 96–97 |
| *Streptococcus gordonii* | 1 | 97 |
| *Streptococcus pyogenes* | 1 | 97 |
| *Streptococcus salivarius* | 1 | 97 |
| *Streptococcus gwangjuense* | 1 | 99 |
| *Streptococcus oralis* | 2 | 97 |
| *Streptococcus mitis* | 1 | 97 |
| *Enterococcus faecalis* | 1 | 97 |
| *Enterococcus faecium* | 2 | 97 |
| *Gemella morbillorum* | 1 | 97 |
| *Lactobacillus iners* | 2 | 97 |
| *Fusobacterium nucleatum* subsp. *Animalis* | 1 | 97 |

*We conducted a BLAST (https://blast.ncbi.nlm.nih.gov) search using Tn*3872*-like integrative conjugative elements (ICE) as a query. Differences in comparison with Tn*3872*-like ICE in ST525 are shown among species that lack the hypothetical protein between ORF20–ORF21.

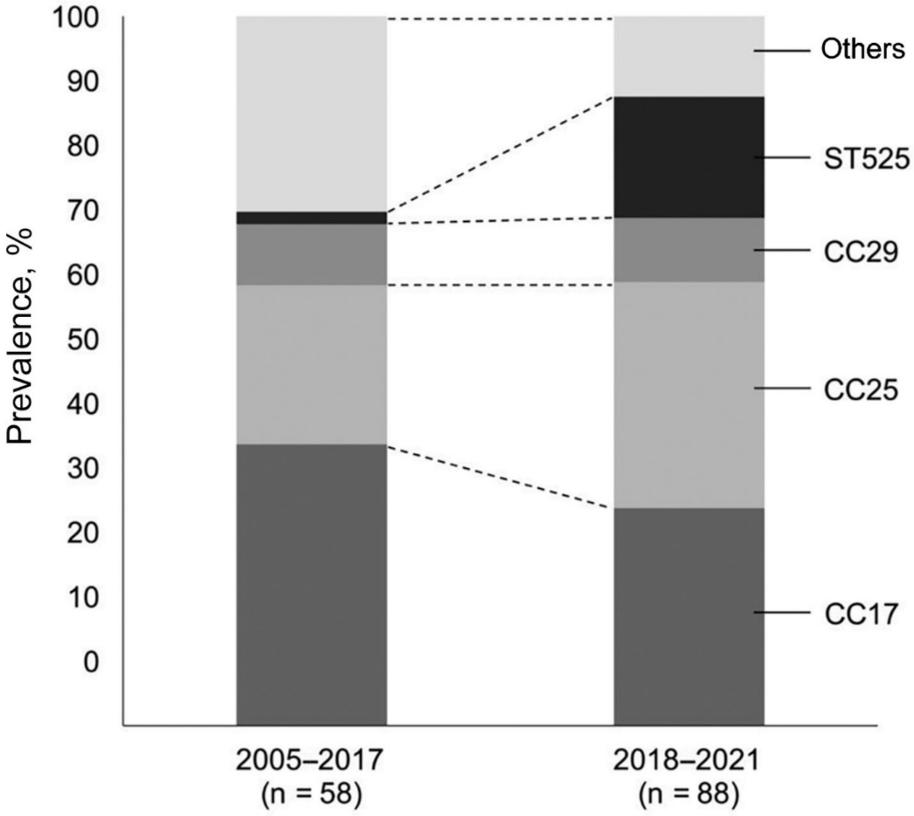


**Appendix 2 Figure.** Temporal changes in the prevalence of CC17, CC25, CC29, ST525, and other clonal complexes and sequence types among *Streptococcus dysgalactiae* subsp. *equisimilis* isolates causing bacteremia, Japan, 2005–2021. CC, clonal complex; ST, sequence type.
